# Pierce's Disease of Grapevines: A Review of Control Strategies and an Outline of an Epidemiological Model

**DOI:** 10.3389/fmicb.2018.02141

**Published:** 2018-09-12

**Authors:** Ifigeneia Kyrkou, Taneli Pusa, Lea Ellegaard-Jensen, Marie-France Sagot, Lars Hestbjerg Hansen

**Affiliations:** ^1^Laboratory of Environmental Microbiology and Biotechnology, Department of Environmental Science, Aarhus University, Roskilde, Denmark; ^2^INRIA Grenoble Rhône-Alpes, Montbonnot-Saint-Martin, France; ^3^Laboratoire de Biométrie et Biologie Évolutive, UMR 5558, CNRS, Université de Lyon, Université Lyon 1, Villeurbanne, France; ^4^Department of Computer, Automatic and Management Engineering, Sapienza University of Rome, Rome, Italy

**Keywords:** *Xylella fastidiosa*, grapevine, Pierce's disease, control strategies, prophylactic, therapeutic, epidemiological model, *Homalodisca vitripennis*

## Abstract

*Xylella fastidiosa* is a notorious plant pathogenic bacterium that represents a threat to crops worldwide. Its subspecies, *Xylella fastidiosa* subsp. *fastidiosa* is the causal agent of Pierce's disease of grapevines. Pierce's disease has presented a serious challenge for the grapevine industry in the United States and turned into an epidemic in Southern California due to the invasion of the insect vector *Homalodisca vitripennis*. In an attempt to minimize the effects of *Xylella fastidiosa* subsp. *fastidiosa* in vineyards, various studies have been developing and testing strategies to prevent the occurrence of Pierce's disease, i.e., prophylactic strategies. Research has also been undertaken to investigate therapeutic strategies to cure vines infected by *Xylella fastidiosa* subsp. *fastidiosa*. This report explicitly reviews all the strategies published to date and specifies their current status. Furthermore, an epidemiological model of *Xylella fastidiosa* subsp. *fastidiosa* is proposed and key parameters for the spread of Pierce's disease deciphered in a sensitivity analysis of all model parameters. Based on these results, it is concluded that future studies should prioritize therapeutic strategies, while investments should only be made in prophylactic strategies that have demonstrated promising results in vineyards.

## Introduction

Pierce's disease (PD) is a disease that affects grapevines (*Vitis vinifera*). The disease is prevalent across the United States (US), from Florida to California, and threatens the country's $30 billion wine industry (Sanscartier et al., 2012). Since the 1880s, PD has caused the decline of more than 35,000 acres of vineyards in Southern California (SC) (Galvez et al., 2010). The aetiological agent of PD is a gram-negative, plant-pathogenic bacterium, *Xylella fastidiosa* subsp. *fastidiosa* (*Xff* ; Davis et al., 1978). The species *X. fastidiosa* (XYLEFA) comprises three well-established subspecies, which infect different plant hosts (Almeida and Nunney, 2015; Marcelletti and Scortichini, 2016), while the existence of a fourth and a fifth subspecies is in dispute (Marcelletti and Scortichini, 2016; Giampetruzzi et al., 2017). Generally, XYLEFA is characterized by a wide plant host range, currently reported to include more than 300 different plant species belonging to 187 genera and 68 families (Stancanelli et al., 2015). While not all of these hosts are susceptible to PD, XYLEFA negatively affects the yields of many economically important crops other than grapevine, such as almond, mulberry, peach, olive, citrus, and plum (Stancanelli et al., 2015). Until recently, the pathogen was primarily found in the US, but in 2013 the first European outbreak was recorded in olive trees in Apulia, Italy (Saponari et al., 2013). Lately, numerous XYLEFA incidents in France and Spain are being reported (Godefroid et al., 2018 and references therein).

XYLEFA is transmitted to host plants by specialized insect vectors (Janse and Obradovic, 2010). All vectors proven to transmit the pathogen belong to the groups of spittlebugs and sharpshooter leafhoppers (Almeida and Nunney, 2015). Specialized XYLEFA vectors can inject the pathogen directly into the xylem of the host plant *via* their sucking mouthparts and as a result of their general feeding habits (Redak et al., 2004). In principle, any xylem-sap feeding insect could be a specialized vector in new areas of entry (Almeida et al., 2005a). Plant hosts susceptible to XYLEFA are often heavily colonized by the pathogen (Hill and Purcell, 1995). However, the mechanisms of specific interactions among XYLEFA subspecies and their susceptible hosts have yet to be resolved (Rapicavoli et al., 2017). Killiny et al. (2013) propose that the production of exopolysaccharides could be a determinative factor of the bacterium's virulence since exopolysaccharides help XYLEFA colonize its two habitats: the foregut of its vectors and the xylem of its plant hosts (Chatterjee et al., 2008). More specifically, XYLEFA exopolysaccharides seem to be responsible for the appearance of water stress symptoms along with plant-derived physiological responses, such as extensive tylose and gum production (Hopkins, 1989; Choi et al., 2013). Examples of plant defense response mechanisms include cell-wall remodeling and upregulation of plant hormone pathways and defense-related recognition receptors (Rodrigues et al., 2013; Giampetruzzi et al., 2016; Zaini et al., 2018), while the various virulence factors of XYLEFA have been extensively reviewed elsewhere (Rapicavoli et al., 2017).

XYLEFA poses a significant threat to susceptible host plants and agriculture worldwide (Almeida and Nunney, 2015). This threat is compounded by the bacterium's high levels of genetic plasticity (Nunney et al., 2014) and the fact that in the first stages of infection the hosts do not display any symptom (Hopkins, 1981; Almeida and Nunney, 2015). The European Food Safety Authority (EFSA) Plant Health Panel has therefore recommended that the European research community intensify studies on the epidemiology and control of XYLEFA (EFSA PLH Panel, 2015a). Several intervention strategies have already been addressed to prevent, suppress, and eliminate the pathogen in vineyards, but as yet there is no strategy available that can cure infected plants in the field (EFSA PLH Panel, 2015a).

This review addresses all the strategies that have been proposed thus far. These strategies are divided into groups A and B. Group A lists only prophylactic strategies, i.e., strategies to avoid infection incidents in vineyards, and Group B includes therapeutic strategies, i.e., strategies suggested for controlling the pathogen and excluding it from an infected vineyard. Tables 1, 2 list all the promising strategies in groups A and B, respectively and include the current status of each strategy and most recent citations. Furthermore, the *Xff* epidemiological model has been developed to describe the disease dynamics of *Xff* in vineyards. The *Xff* epidemiological model identifies key parameters in the spread of PD that should be pursued by current and future intervention strategies. The framework of this model may assist the future design of more complex models of *Xff* and XYLEFA disease dynamics.

**Table 1:** Summary of promising strategies to prevent infection with *Xff* in vineyards (group A).

**Strategy**	**Promising case studies**	**Status of strategy**	**Latest citations**
Control of insect vectors	Insecticides (esp. neocotinoids)	Applied in vineyards and nurseries	Daugherty et al., 2015; Redak et al., 2017
	Kaolin-based products	Tested in the vineyard	Tubajika et al., 2007
	Screen barrier	Tested in the vineyard	Blua et al., 2005
	Mimetic insecticidal peptides	Under development	Federici, 2005
	Paratransgenesis with E325	Tested *in vivo* (GWSS)	Arora, 2015
	Virus HoCV-1	Tested *in vivo* (GWSS)	Hunnicutt et al., 2008; Falk et al., 2014; Kamita et al., 2016
	Fungus *I. poprawskii*	Tested *in vivo* (GWSS)	Cabanillas and Jones, 2013
	Egg parasitoids (esp. *G. ashmeadi*)	Tested in California and French Polynesia	Grandgirard et al., 2008; CDFA, 2016
Control of non-vine host plants and vine propagation material	Removing wild plant hosts	Applied in vineyards	EFSA PLH Panel, 2015a
	Sanitating *via* long-duration HWT	Applied in nurseries	Goheen et al., 1973; EFSA PLH Panel, 2015b
Alteration to cropping techniques	Roguing vines with persistent PD	Applied in vineyards	Sisterson and Stenger, 2013
	Regulated deficit irrigation	Tested in the vineyard	Krugner and Backus, 2014
Breeding or engineering PD-resistant/tolerant *V. vinifera*	N18-6 × Flame Seedless lines	Tested in the greenhouse	Lin et al., 2017
	Hybrid vines with gene PdR1b	Tested in the vineyard	Walker and Tenscher, 2017
	Rootstock TS-50 expressing pPGIP	Tested in the vineyard	Dandekar et al., 2017
	Rootstocks expressing CAP	Tested in the vineyard	Dandekar et al., 2017
	VvPR1 and UT456 transgenes	Tested in the vineyard	Lindow et al., 2014
	Vines expressing RpfF	Tested in the vineyard	Lindow et al., 2014
Control *via* avirulent XYLEFA strains	Inoculating with strain EB92-1	Tested in the vineyard	Hopkins et al., 2015
	Inoculating with strain ΔPD1311	Tested in the greenhouse	Burr et al., 2017
Control *via* other beneficial bacteria and fungi	endophytic fungi and bacteria	Tested in the greenhouse	Rolshausen and Roper, 2016; Rolshausen et al., 2017
	DSF-producing/inhibitory strains	Tested in the greenhouse	Lindow et al., 2005

**Table 2:** Summary of promising strategies to exclude *Xff* from an infected vineyard (group B).

**Strategy**	**Promising case studies**	**Status of strategy**	**Latest citations**
Bacteriophage cocktails	Lytic phages Sano, Salvo, Paz & Prado	Tested in the greenhouse	Ahern et al., 2014; Das et al., 2015
Antagonistic bacteria	Inoculating with *P. phytofirmans* strain PsJN	Tested in the greenhouse	Lindow et al., 2015, 2017a
Natural, antibacterial substances	Radicinin	Tested *in vitro*	Rolshausen et al., 2015, 2017
	Antibiotics tetracycline, gentamicin, ampicillin, kanamycin, novobiocin, chloramphenicol and rifampin	Tested *in vitro*	Kuzina et al., 2006
	Inoculating with antibiotic streptomycin	Tested in the greenhouse	Kirkpatrick et al., 2004
	Antimicrobial peptides PGQ, indolicidin, magainin 2, and dermaseptin	Tested *in vitro*	Kuzina et al., 2006
	12 phenolic compounds (esp. catechol, caffeic acid and resveratrol)	Tested *in vitro*	Maddox et al., 2010
	Inoculating with microelement zinc sulfate/oxide	Tested in the greenhouse	Kirkpatrick et al., 2004
Other defense-stimulating compounds	Application of ABA by foliar sprays or soil drenches	Tested in the greenhouse	Meyer and Kirkpatrick, 2011
	Iron chelators lactoferrin, EDTA (ethylenediaminetetraacetic acid) and EDDS	Tested *in vitro*	Koh and Toney, 2005

## Group A: prophylactic strategies

**Figure 1:**
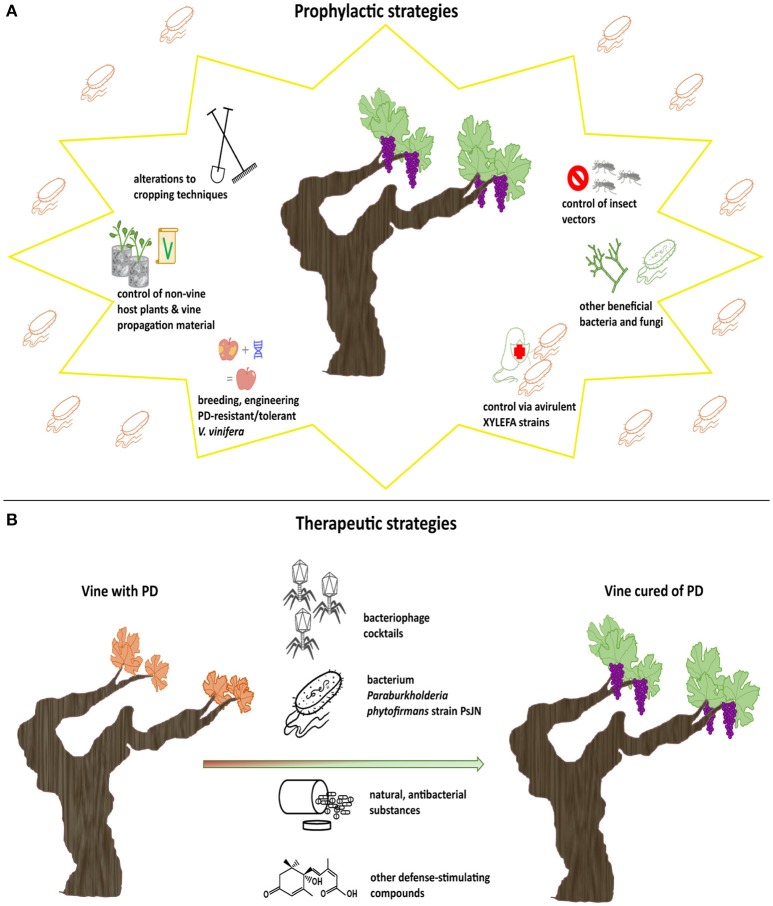
Summary of all types of prophylactic and therapeutic strategies against PD presented in this paper. Prophylactic strategies address healthy vines in an attempt to prevent PD **(A)**, while therapeutic strategies address *Xff*-infected vines in order to cure them of PD **(B)**.

Prophylactic practices against PD are typically aimed at either inoculum reservoirs or modes of dispersal of the pathogen in vineyards, i.e., host plants and insect vectors (Almeida and Purcell, 2003). When prophylactic measures deal with alterations to cropping techniques, a combination of practices is highly recommended for optimal results (EFSA, 2016). A summary of all types of prophylactic strategies discussed below is illustrated in Figure 1A.

### Control of insect vectors

Maintaining the insect vector populations at low levels has traditionally been attempted *via* foliar and soil-applied insecticide treatments (Almeida et al., 2005a; EFSA, 2015). Vine-growers have often combined insecticides with a specific type of insect traps, known as yellow sticky traps, either to indicate the necessity for or monitor the efficiency of insecticides in the field (EFSA, 2016). Moreover, in Californian nurseries citrus plants have typically been fumigated with insecticides (Ancona et al., 2010). The basic vector of *Xff* in vineyards is the glassy-winged sharpshooter [*Homalodisca vitripennis*, formerly known as *H. coagulata* (GWSS)] (Hopkins and Purcell, 2002). Given that the vast majority of control strategies against *Xff* vectors aim at GWSS, this review focuses on GWSS exclusively. Many studies have tracked the susceptibility of GWSS to different classes of insecticides, in particular neocotinoids, butenolides, pyrethroids, carbamates, and organophosphori (e.g., Bextine et al., 2004a; Prabhaker et al., 2006; Tubajika et al., 2007). Imidacloprid, a systemic neocotinoid, has proven most efficient against GWSS due to its persistence in the plant tissue, its systemic activity and its selectivity for xylem and phloem-feeding insects (Daugherty et al., 2015). Regardless of its apparent success, imidacloprid has adverse effects on bee health and it is going to be banned within the European Union by the end of 2018 (EFSA, 2018).

A recent report on systematic monitoring for insecticide resistance in GWSS has detected a pattern of decreasing susceptibility in the vector to most insecticides applied in the last 14 years (Perring et al., 2016, 2017). Nonetheless, the higher flow of GWSS recorded in 2017 does not appear to be due to the development of resistance to insecticides (Perring et al., 2017). Nevertheless, given the prolonged employment of insecticides, resistance development in the target insects is to be expected in addition to environmental contamination with insecticide residues and effects on non-target organisms (Pimentel, 2005). Despite those issues, insecticides continue to play a central role in the control of PD, especially in the case of commercial nurseries (Redak et al., 2017). Vine-growers often carry out within-vineyard sprays to supplement coordinated, area-wide insecticide sprays (Rapicavoli et al., 2017).

Three alternatives to insecticides for GWSS have already been described however. The first is Surround® WP (Engelhard Corporation, Iselin, New Jersey, US), a kaolin-based product. Vineyard trials showed that coating vines with white kaolin both sabotages GWSS preference for grapevine and increases GWSS mortality after 48 h' exposure (Wood and McBride, 2001; Tubajika et al., 2007). The second alternative is harpin, a protein-based elicitor that induces the hypersensitive response of plants and is produced by certain phytopathogenic bacteria. Harpin was included in the field study by Tubajika et al. (2007). Similar to kaolin, the results of harpin applications in the field suggest that vines treated with this protein have a lower chance of developing PD. Nevertheless, harpin appears to be less lethal to GWSS compared to kaolin. The third alternative is a unique tactic tested by Blua et al. (2005). In order to physically impede GWSS from entering a field, the researchers assessed the impact of a 5-m-high screen barrier on GWSS dispersal and demonstrated that only 6% of GWSS released outside the barrier were able to fly over it and enter the field.

In addition to the above-mentioned conventional methods, there are various organic approaches to control the spread of XYLEFA vectors. One example is mimetic technology, which involves the development of peptides that closely resemble the natural substrates of proteins vital for the survival of an unwanted pest organism. The host organism is then engineered to express the mimetic peptide, which is lethal to the pest. With regard to PD, mimetic insecticidal peptides have been produced in order to control GWSS (Federici, 2004). The aim was to make peptides that could bind to transport proteins in the GWSS midgut microvillar membrane or to GWSS salivary enzymes. The best candidate target so far is V-ATPase c, a protein that affects H+/K+ transport, which in turn maintains insect gut pH (Federici, 2005). Tests are still ongoing to ensure the efficiency of the constructed antibody peptides. Long-term plans include the expression of any successful peptides in transgenic vines (Federici, 2005).

Another example is paratransgenic control or paratransgenesis. Paratransgenesis utilizes either genetically modified symbionts or commensal bacteria as vehicles of delivery of anti-pathogen molecules within the insect gut. Subsequently, these molecules block any transmission of the bacteria concerned (Hurwitz et al., 2011). With respect to PD control, more than one bacterial candidate has been investigated. In one case, the commensal *Pantoea agglomerans* E325 was chosen, since it is able to persist within the foregut of GWSS (Arora, 2015) and deliver anti-*Xylella* effector proteins (Lampe et al., 2007). *P. agglomerans* E325 has been engineered to express melittin and a scorpine-like molecule, two antimicrobials that are toxic for XYLEFA (Arora, 2015). The manipulated GWSS were less capable of carrying the pathogen than the control GWSS and failed to infect naïve grapevines with *Xff* (Arora, 2015). In another case, a bacterial symbiont of GWSS that can survive within vines, *Alcaligenes xylosoxidans denitrificans*, was selected as the vehicle of delivery (Miller et al., 2001; Lauzon et al., 2003). The latest reports confirm that *A. xylosoxidans denitrificans* survives in GWSS for just 5 weeks (Bextine et al., 2004b; Ramirez et al., 2008). RNA interference also has temporary effects. Falk et al. (2007) investigated various mRNAs that, if blocked by RNA interference, would kill or reduce the survival and/or fecundity of GWSS. The final report concluded that no *in vitro* feeding or transgenic vine essays with RNA-interference-inducers could have consistent phenotypic expression on exposed GWSS (Falk et al., 2016).

Besides biotechnological efforts, naturally occurring parasites have been considered in the “organic combat” against PD. The treatment of PD *via* augmentation of natural enemy populations of its insect vectors has generally been a very popular approach. Hunter et al. (2004) examined the potential of a whitefly iridovirus (WFIV). By infecting GWSS with WFIV *via* microinjection and sprays, they succeeded in negatively impacting the vector's fecundity and longevity. They also observed that adult GWSS that acquired the iridovirus could pass it on to other healthy individuals and perhaps lay infected eggs. Following that, Hunnicutt et al. (2006) isolated an ssRNA virus, *Homalodisca coagulata* virus-1 (HoCV-1) after detecting sequences of the virus in expressed sequence tag libraries from GWSS. HoCV-1 was able to establish infection regardless of the host plant species or the developmental stage and sex of GWSS, and its host range extended to other sharpshooter species (Hunnicutt et al., 2008). Although, infections did not always lead to acute mortality, HoCV-1 coupled with other preventive strategies or engineered to deliver toxic peptides and/or induce systemic RNA interference (RNAi) was able to curb GWSS populations significantly (Bextine et al., 2009). Such combination of strategies is presently being explored (Falk et al., 2014; Kamita et al., 2016). Along with HoCV-1, GWSS harbors a dsRNA virus of the genus *Phytoreovirus* designated as *H. vitripennis* reovirus. Even though *H. vitripennis* reovirus is widely distributed among GWSS populations in the US, it has caused no detectable decline in them, thus the contribution of this virus to the biocontrol of GWSS is questionable (Stenger et al., 2010).

Other parasites with the potential to regulate GWSS are entomopathogenic fungi, but current knowledge about these fungi is limited. Kanga et al. (2004) suggest that the fungi *Pseudogibellula formicarum* and *Metarhizium anisopliae* are likely candidates for the biocontrol of GWSS. Three more fungal candidates have subsequently been added to the list of potential biocontrols: *Hirsutella homalodiscae, Sporothrix* spp. and *Beauveria bassiana* (Boucias et al., 2007; Dara et al., 2008; Lietze et al., 2011). The latest case study refers to the fungus *Isaria poprawskii* and investigated its pathogenicity to GWSS (Cabanillas and Jones, 2013). *In vitro* trials showed that the fungus is virulent and pathogenic to GWSS nymphs and adults. Ultimately, the study deemed *I. poprawskii* to be a promising biocontrol agent of GWSS because it can cause natural epizootics, has a spectrum that encompasses more than one *Xff* insect vectors, is heat-tolerant, and is easy to culture. Surprisingly, the potential of entomopathogenic bacteria against GWSS, such as the widely-applied *Bacillus thurigiensis*, remains unexplored and should be addressed.

In addition to viral and microbial enemies, the utility of other GWSS enemies has been investigated. Fournier et al. (2004) embarked on a large-scale project with the aim of determining any important predators during the various life stages of GWSS. The project screened the gut concent of GWSS associated predators by egg-specific monoclonal antibody-based, enzyme-linked immunosorbent assays (ELISA), and by PCR assays using GWSS-specific mitochondrial primers. As many as 1,578 field-collected spider and insect species were identified as predators (Hagler et al., 2013). Results indicated that GWSSs, at any life stage, are not the main prey for the generalist predators examined. No other potential predators, such as birds, have been studied, and a subsequent study has corroborated the finding that arthropod predators may not be useful in GWSS regulation (Daugherty et al., 2015). In addition, multiple egg parasitoids of GWSS have been identified. Triapitsyn (2013) has produced an exhaustive review that lists more than 20 egg parasitoids of GWSS. A mymarid egg parasitoid, *Gonatocerus ashmeadi*, has successfully been used for the biocontrol of GWSS in French Polynesia and, consequently, released with *G. morgani and G.morrilli* in California (Grandgirard et al., 2008, 2009; CDFA, 2016).

### Control of non-vine host plants and vine propagation material

*Xylella fastidiosa* subsp. *fastidiosa* has a very broad host range that includes common cultivars, as well as wild herbs and weeds (Purcell and Saunders, 1999; Wistrom and Purcell, 2005; Stancanelli et al., 2015). There have been various reports on host plants that pose a threat to vineyards (Purcell and Saunders, 1999; Costa et al., 2004; Wistrom and Purcell, 2005; Sisterson et al., 2010). Removal of these undesirable plants that serve as reservoirs for the bacterium or its vectors currently constitutes a major preventive strategy in the field (EFSA PLH Panel, 2015a). In addition, vineyards should be distanced from non-vine plants that can harbor nymphal and adult stages of *Xff* vectors, such as citrus, alfalfa, almond, maple, cherry, Spanish broom, and western redbud (Janse and Obradovic, 2010; Sisterson et al., 2010). Even if non-vine host plants are not nececcarily an important source of inocula, they may still serve as a pool of vectors (Sisterson et al., 2010). For example, GWSS frequently overwinters in citrus groves located next to vineyards in California (Almeida et al., 2005b). The chances of PD occurring in pest-free areas could be reduced by prohibiting the import of host plants that represent important cultivars (EFSA PLH Panel, 2015a). Furthermore, planting certified nursery plants and growth plants under exclusion conditions (screen-house and greenhouse) could prevent the spread of PD (EFSA PLH Panel, 2015a).

Hot water treatment (HWT) has been the most popular approach to avoid PD occurrence in nurseries, (Goheen et al., 1973; EFSA PLH Panel, 2015b). There are two different protocols of HWT: a short-duration HWT and a long-duration one (Waite and Morton, 2007). In order to eliminate any *Xff* infection, grapevine planting material is exposed to long-duration HWT (50°C for 45 min; EFSA PLH Panel, 2015b). The planting material can either be vine cuttings or 1-year-old rooted vines. In both instances, it is of vital importance that the planting material is fully dormant otherwise it could be irreparably damaged (Waite and Morton, 2007 and references therein). That said, some varietal-based sensitivity to HWT does exist and is expressed as slower establishment of the treated cuttings in the field (Waite and Morton, 2007 and references therein). Additionally, the success of such treatment can depend upon many pre- and post-HWT propagation practices. For example, long-term cold storage of treated material that has not been properly soaked or ventilated can cause irreparable damages to the plant (Gramaje et al., 2014 and references therein).

### Alterations to cropping techniques

Cropping techniques are crucial in the attenuation of plant disease risk (Palti, 1981). Specific practices have been recommended to avoid the occurrence or diminish the spread of PD in vineyards. One example is soil and vegetation management, such as regular rototilling and hand weeding to repress nymphal populations of insect vectors that thrive on herbaceous hosts (EFSA, 2016). Regular summer and autumn pruning of symptomless vines might also eliminate PD infections (Hopkins and Purcell, 2002), albeit posing a risk of *Xff* mechanical transmission should pruning tools not be regularly disinfected (Krell et al., 2007; Overall and Rebek, 2015). Indeed, severe pruning might stop *Xff* from reaching the grapevine's permanent structure and establishing a chronic infection (Hopkins and Purcell, 2002). Nevertheless, severe pruning does not significantly contribute to the recovery of vines infected by *Xff* and should be viewed as a disease strategy of minor efficacy (Daugherty et al., 2018). During the dormant season, entire vines that either have PD symptoms for more than one year or extensive foliar symptoms on most canes and with tip dieback of canes for less than one year must be removed. Such vines are unlikely to recover or produce a significant crop, and roguing them would prevent the rapid spread of PD to adjacent vines (Sisterson and Stenger, 2013). Specific destruction protocols toward a better management of infested plant residues and tools are still pending, but some examples for XYLEFA involve autoclaving and incineration (Onghia et al., 2017).

Another measure is regulated deficit irrigation. In a study comparing fully irrigated plants to water-stressed ones, Krugner and Backus (2014) argue that GWSS avoid water-stressed plants and spend shorter periods ingesting their xylem fluid. Thus, provided the grower is aware of the drought-sensitive growth stages of the grapevine varieties, this strategy could be sufficiently effective (Krugner and Backus, 2014). The importance of low plant water content has previously been underlined by Boisseranc (2010). In this study, Boisseranc identified high levels of soil dryness, soil sulfur content, vine calcium, and boron content as significantly reducing the predicted risk of PD. Recently, additional variables that negatively correlate to increased PD incidence have been pinpointed, of which cation exchange capacity and low soil pH appear to be the most significant (Costello et al., 2017). All the above-mentioned plant and field variables should be considered and potentially incorporated in the regular management of vineyards to control PD.

### Breeding or engineering PD-resistant/tolerant *V. vinifera*

In grapevine, *V. vinifera* cultivars are susceptible to PD, while resistance is found in other species that are not of sufficiently high quality for wine production. Thus wild, resistant *Vitis spp*. have been used in traditional plant breeding experiments with elite cultivars. Lin et al. (2017) report that 15 out of 183 hybrid lines of N18-6 crossed with Flame Seedless developed few to no PD symptoms in greenhouse trials. It is worth noting that N18-6 comes from the cross of DC1-56 (W1521 x Aurelia) x Orlando Seedless (D4-176 x F9-68) and is characterized by a high yield, good flesh texture and flavor. Moreover, N18-6 has been able to survive under high PD pressure in the field. The study is ongoing and is currently focusing on identifying the genetic source of resistance.

Another study, described by Walker and Tenscher (2017), has discovered the PD resistance gene *PdR1* (chromosome 14) in *V. arizonica/candicans* line b43-17 (alleles *PdR1a, PdR1b*) and *V. arizonica* line b40-14 (allele *PdR1c*). By backcrossing elite grapevine cultivars with the PdR1b gene they succeeded in reaching progeny of 97% *V. vinifera*. They then refined their selection by evaluating fruit and vine characteristics, performing resistance screenings in the greenhouse, assessing the wine in various wine tastings, and finally establishing field trials. Their most resistant varieties were grown in the field at various PD hotspots in California during a 16-year field trial and have yet to show any PD symptoms. Walker and Tenscher's project, which has been running since 2001, has produced 19 PD-resistant scions and three PD-resistant rootstocks. Research is ongoing to investigate the possibility of breeding PD resistance to grape varieties *via* other means, such as gene *PdR2* (chromosome 8) of *V. arizonica/girdiana* line b42-26, and *V. shuttleworthii* (Walker and Tenscher, 2017).

A major offshoot of Walker and Tenscher's project has since been initiated (Walker et al., 2014). One of the scopes was to identify more resistance genes that map to regions different to *PdR1* (i.e., other than chromosome 14), thus allowing resistance sources to be combined to produce breeding lines of wider resistance to PD (Walker et al., 2014). Five additional resistant accessions, T03-16, ANU67, b41-13, b47-32, and A14, were singled out after screening many different *Vitis* species. Accession b46-43 was also tested, but ultimately discarded since the mapping results located its resistance source on chromosome 14, the same as for *PdR1* (Walker et al., 2017). The mapping experiments are still underway in order to elucidate the origins of the resistance in the rest of the accessions. To date chromosome 14 has been ruled out as a possibility.

Other than for breeding purposes, different transgenic lines of *V. vinifera* have been produced to protect grapevines from *Xff*. For example, Kirkpatrick et al. (2012) produced Hxf-expressing grapevines, i.e., transgenic grapevine lines that are able to express a Hxf protein in their xylem fluid. The research was based on an earlier finding that Hxf proteins (hemagglutinins) are involved in XYLEFA virulence and cell-to-cell aggregation (Guilhabert and Kirkpatrick, 2005). The conclusion of the study and field trials of transgenic Hxf-expressing grapevines was that even though the Hfx gene seems to delay the spread of XYLEFA, it is unable to provide long-term protection against the bacterium (Gilchrist and Lincoln, 2016).

In a different study, *Vitis vinifera* cvs. “Thompson Seedless” (TS) and *Vitis vinifera* cvs. “Chardonnay” (CH) were transformed to express the pear fruit PGIP-encoding gene (*pPGIP*) (Agüero et al., 2005). PGIPs (PG-inhibiting proteins) are produced by plants to block cell wall-degrading enzymes (Esquerré-Tugayé et al., 2000). The latter are typical effector proteins of plant pathogens (Esquerré-Tugayé et al., 2000). XYLEFA's arsenal includes a polygalacturonase (*Xf* PG), which digests cell wall pectin, and several β-1,4-endo-glucanases (EGase), which digest xyloglucan polymers (Roper et al., 2007). Thus, if allowed to express *Xf* PG and EGase the bacterium can move around neighboring xylem vessels by degrading the pit membranes separating those vessels (Harakava et al., 2001). This process releases nutrients for the pathogen and eventually leads to vessel occlusion and vine death (Harakava et al., 2001). Therefore, lines with high (TS-50, TS-74, TS-201, CH-329, and CH-330), moderate (TS-56, TS-188, CH-168, and CH-327) and low (CH-248 and CH-251) PGIP activity were initially trialed by inoculating the vines with *Xff* under greenhouse conditions. *pPGIP* succeeded in protecting three lines (TS-50, CH-329, and CH-327) against PD, as these developed only mild symptoms of the disease (Harakava et al., 2001). Finally, Dandekar et al. (2010) selected line TS-50 to establish a seven-year-long field evaluation. For that study, four different constructs of *pPGIP-*plants were planted in two different PD hotspot locations, either as own-rooted plants or as rootstocks, and were mechanically inoculated with *Xff* (Dandekar et al., 2010). The results of the study supported the hypothesis that TS-50 rootstocks can sufficiently protect the scion from PD (Dandekar et al., 2017).

Plants have developed a first line of defense against invaders, the innate immune response, to detect pathogens and activate defense responses (Janeway and Medzhitov, 2002). The innate response is not always successful, but Dandekar et al. (2012) hypothesized that if two different types of it are combined in one construct they will manage to efficiently block a pathogen. To exclude *Xff*, they constructed a chimeric antimicrobial protein by joining human neutrophil elastase (HNE), a recognition protein, and cecropin B (CB), a lytic peptide (Gupta, 2008). HNE has the advantage of cleaving the most abundant and accessible protein in *Xff* membrane, protein MopB (Dandekar et al., 2012). Moreover, the interaction between CB and HNE would promote both cleavage (HNE) and lysis (CB) of *Xff* (Dandekar et al., 2012). Later, the construct was reformed to comply with US regulations on genetically modified organisms. HNE was replaced by P14a from *V. shuttleworthii*, which, like HNE, functions as a serine protease, and CB was replaced by HAT52 and/or PPC20 (Dandekar et al., 2017 and references therein). New constructs and a combination of old and new constructs have been used to transform vines for the 101-14 and 1103 rootstocks (Dandekar et al., 2014). The newly produced transgenic vines are being tested in the greenhouse and in the field (Dandekar et al., 2017). Previous field trials for the old construct (HNE-CB) expressed in a TS rootstock and manually inoculated with *Xff* have been very promising (Dandekar et al., 2017).

Efforts to produce transgenic vines that are resistant to *Xff* have generally been a favored approach in various studies. For example, vines have been engineered to express gene *rpfF* for a diffusible signal factor (DSF) synthase RpfF that originates from XYLEFA (Lindow et al., 2010; Beaulieu et al., 2013). The *rpfF* gene is thus essential for the production of DSF that in turn upregulates factors required for biofilm formation but represses traits necessary for plant colonization (Newman et al., 2004). Therefore, a high concentration of DSF moleculesin transgenic vines could be expected to change the quorum sensing of *Xff* and thereby increase attachment and aggregation, and limit systemic spread *via* the xylem (Lindow et al., 2010). DSF-producing transgenic “Freedom” vines were tested in the field and indeed reported to be two to fourfold more resilient to PD after natural or mechanical inoculation with *Xff* (Lindow et al., 2014). Presently, the DSF project is undergoing various improvements to enhance the effect of DSF and reduce costs (Lindow et al., 2017b). These involve the application of fatty acids that are homologous to DSF, transformation of many different vine varieties and rootstocks, as well as the use of a variant of gene *rpfF*, which encodes a protein with sequences that can direct the enzyme to the chloroplast (Lindow et al., 2017b). Interestingly, similar efforts are in progress to transiently express the *rpfF* gene of the olive-infecting XYLEFA strain CoDiRO *in planta* using a TMV-based vector (D'Attoma et al., 2017).

Another project dealt with with the generation of transgenic vines that could suppress apoptosis and programmed cell death (PCD), thus rendering the transgenes asymptomatic hosts. Specifically, (Gilchrist et al., 2006) performed functional cDNA screening to identify anti-PCD genes expressed in grapevines, which yielded 12 genes. Transgenic “Freedom” and TS vines over-expressing a variety of those 12 genes were inoculated under greenhouse conditions and were later shown to harbor *Xff* concentrations comparable to those in resistant grapevines (Gilchrist et al., 2007; Gilchrist and Lincoln, 2008). Finally, two DNA transgenic sequences, VvPR1 and UT456, were selected as the most effective ones (Gilchrist and Lincoln, 2011). Between 2012 and 2014, transgenic rootstocks of VvPR1 and UT456 were evaluated in two field sites, under both artificial and natural *Xff* pressure, and found to efficiently protect TS scions against PD (Gilchrist and Lincoln, 2014).

In an effort to provide longer-lasting and more robust protection against PD, Gilchrist et al. (2014) commenced a project with the aim of combining all DNA constructs mentioned in section Breeding or Engineering PD-Resistant/Tolerant *V. vinifera* in pairs. The combination of transgenes takes place in the rootstock, which was being tested for its efficacy of cross-protecting the susceptible scions 1103 and 101-14 (Gilchrist et al., 2014). Dual construct transgenic plants are currently being mechanically inoculated with *Xff* and monitored under greenhouse conditions (Gilchrist et al., 2017). Field experiments were planned for spring 2018 and the long-term objective of the project is to combine more than two constructs in a gene (Gilchrist et al., 2017).

### Control via avirulent XYLEFA strains

An early report on naturally occurring benign and weakly virulent strains of XYLEFA that could shield susceptible grapevines against *Xff* can be found in Hopkins (1992). The hypothesis was that these strains cross-protect susceptible vines and induce systemic acquired resistance if inoculated prior to an infection with a highly virulent *Xff* (Hopkins, 1992). In 2005, an update on the same project expanded the potential of avirulent and benign strains (Hopkins, 2005), involving six such strains. Strains PD-1, PD91-2, PD94-1, and PD95-6 were weakly virulent and strains Syc86-1 and EB92-1 were avirulent to grapevines (Hopkins, 2005). The trials took place both in the greenhouse (6 months) and in three different vineyards (2 years) and involved three different cultivars. Under both conditions, only individuals treated with strain EB92-1 seemed to develop few to no symptoms of PD compared to vines treated with the other five strains (Hopkins, 2005). Strain EB92-1 was subsequently selected for further testing in the field. After a four-year field trial on Pinot Noir and Cabernet Sauvignon cultivars, the results corroborated the finding that EB92-1 can efficiently protect those cultivars against *Xff* under heavy disease pressure conditions (Hopkins et al., 2015).

Likewise, Burr et al. (2013) created the *Xff* disruption strain ΔPD1311 by deleting gene PD1311. PD1311 had previously been discovered to encode a putative acyl-CoA synthetase, and the deletion of this gene was found to render *Xff* non-pathogenic (Burr et al., 2013). Further investigation into the role of PD1311 demonstrated that it participates in biofilm formation and aggregation, and influences motility (Burr et al., 2014). The value of strain ΔPD1311 as a biocontrol agent was also investigated because ΔPD1311 appeared to be avirulent (Burr et al., 2015) and capable of reducing the virulence of *Xff in planta* (Burr et al., 2014). By the time the greenhouse inoculation experiments had been concluded, the suitability of strain ΔPD1311 as a biocontrol agent against PD was confirmed and the need for field tests underlined (Burr et al., 2017). Such an approach seems promising and similar studies are underway (Lin and Shi, 2017), although the risk that the avirulent strains may revert to virulent by horizontal gene transfer from *Xff* should be taken into account.

### Control via other beneficial bacteria and fungi

Plants are naturally colonized by specific beneficial microbes such as plant growth-promoting rhizobacteria and mycorrhizal fungi (Van Wees et al., 2008). This microflora promotes plant health by various mechanisms, including the production of antagonistic substances and induction of systemic resistance against pathogens (ISR) (Van Wees et al., 2008; Beneduzi et al., 2012). This principle has prompted the search for potential beneficial bacteria and fungi to stop *Xff* infections of susceptible hosts. The first study on plant endophytes antagonistic toward *Xff* described 19 absolute inhibitor strains belonging to six different *Bacillus* RFLP groups, *Cellulomonas* sp., *Rahnella* sp., and *Streptomyces* sp. (Kirkpatrick et al., 2003). Unfortunately, *in planta* greenhouse trials using five of these isolates showed that they cannot prevent initial *Xff* infections (Kirkpatrick et al., 2003).

A follow-up study assessed the six isolates by establishing a field trial. This revealed that two isolates, *Cellulomonas* sp. and a *Bacillus* sp. strain, had great potential to suppress *Xff* (Kirkpatrick et al., 2005). Subsequently, the putative *Cellulomonas* sp. strain was sequenced and identified as a *Bacillus subtilis* strain (Kirkpatrick and Wilhelm, 2006). Unfortunately, continuation of the field trials showed that the *Bacillus subtilis* strain and the other *Bacillus* sp. strain are unable to provide long-term protection to the inoculated vines (Kirkpatrick and Wilhelm, 2007). Other isolates belonging to the genera *Pseudomonas, Kocuria, Bacillus, Curtobacterium, Pantoea, Paenibacillus, Stenotrophomonas* exhibited a promising inhibition of *Xff* during *in vitro* screenings, but subsequent field trials failed due to inadequate inoculations (Kirkpatrick and Wilhelm, 2006, 2007). All in all, a significant vine endophyte library has been created by this group. Rolshausen et al. (2017) took the next step and showed successful reduction of PD symptoms by *Pseudomonas* sp. and *Achromobacter* sp. These two bacteria were strongly colonizing vines that have escaped PD under great disease pressure and were tested by introducing them *in planta* via needle inoculation of shoots or vacuum infiltration of vines before the rooting stage.

XYLEFA produces a type of “xanthan gum,” which is suggested to enable the pathogen to adhere, aggregate and ultimately clog the xylem vessels (Lee and Cooksey, 2004). A study on xanthan-gum development by XYLEFA has characterized an endophytic bacterium with high gum-degrading activity, *Acinetobacter johnsonii* strain GX123 (Cooksey and Schiller, 2003). The advantage of this isolate is that unlike other endophytes (Hardoim et al., 2008) it does not display any plant cellulose-degrading activity, making it safe for *in planta* tests (Cooksey and Schiller, 2003). Greenhouse tests on oleander plants evinced that the endophyte can reach a higher titer in the plants and spread faster and further from the inoculation site when it is co-inoculated with *Xff* (Cooksey and Schiller, 2003). A year later, all the inoculated plants displayed PD symptoms, but the symptoms of those co-inoculated with *A. johnsonii* GX123 were less severe (Lee and Cooksey, 2004). The results suggest strain GX123 is a promising biocontrol tool, although more tests are needed to confirm the efficiency of this gum-degrader in vines.

Another virulence factor of *Xff* targeted by researchers through endophytes is DSF. The role of DSF has already been described above. During a 5-year study, Lindow and his collaborators exploited the role of DSF to highjack cell-cell communication in XYLEFA. Among others, they collected grapevines from PD affected vineyards and tomato and cruciferous crop plants infected with pathogens *Xanthomonas campestris* pv. *vessicatoria* and *X. campestris* pv. *campestris* (Lindow et al., 2004). In all, they unraveled two types of disturbance of the cell-cell signaling by the isolates: DSF degradation and another unidentified mode of DSF inhibition (Lindow et al., 2004). They also isolated endophytes that can produce DSF (Lindow et al., 2004). The DSF-producing strains were all *Xanthomonas spp*., while the DSF-inhibitory ones belonged to the genera *Paenibacillus, Pseudomonas, Staphylococcus*, and *Bacillus* (Lindow et al., 2004). To test the potential of all isolate categories, they co-inoculated vines with *Xff* plus either the DSF-producing or the DSF-inhibitory isolates in the greenhouse. In spite of their insufficient systemic spread in the vine, all categories reduced PD symptoms, with DSF-producing isolates appearing to perform slightly better than the DSF-inhibitory isolates (Lindow et al., 2004). Thus, all the isolates proved very promising as protective agents for grapevines against *Xff* (Lindow et al., 2005).

Finally, fungal endophytes have also been considered for the protection of vines against PD. First, Rolshausen et al. (2010) isolated endophytic fungi from PD-escape vines. Later, *in vitro* studies revealed seven grapevine endophytic fungi, which can significantly inhibit XYLEFA (Rolshausen and Roper, 2016). The fungi belong to the genera *Aspergillus, Cochliobolus, Cryptococcus, Discostroma, Geomyces, Phaeosphaeria*, and *Ulocladium* (Rolshausen and Roper, 2016). *In planta* bioassays showed that *Geomyces* sp., *Cochliobolus* sp., and *Cryptococcus* sp. are able to mitigate PD symptoms under greenhouse conditions (Rolshausen and Roper, 2016; Rolshausen et al., 2017). Lastly, the research group investigated the healing potential of natural compounds produced by some of those fungi (further details can be found in the “therapeutic strategies” section below)^1^.

## Group B: therapeutic strategies

Curative strategies are crucial for the successful protection of vines against *Xff*. These strategies are characterized by the fact that they directly target susceptible plants and can both protect and cure sick vines by eliminating *Xff*. Most therapeutic strategy mechanisms rely heavily on biological control agents, although some exceptions are also reported. Thus, most of the strategies presented below have the advantages of being environmentally friendly, specific and cost effective. Some of them are also based on natural enemies of *Xff*, which minimizes their ecological impact. This is because *Xff* 's natural enemies are normally found only where *Xff* is present and are therefore unable to persist to an ecosystem without it and become invasive. A summary of all types of therapeutic strategies reviewed below is depicted in Figure 1B.

### Bacteriophages of *Xff*

Bacteriophages (phages) are viruses that infect bacteria. Phages are classified into the virulent or the temperate group based on their lifestyle. Phages in the virulent group follow a lytic lifestyle, i.e., they destroy the bacterial cells they infect. A given phage species is capable of infecting one bacterial species with a high degree of specificity, usually in the strain level. Owing to this specificity, different virulent phages are chosen and applied in a cocktail to treat bacterial infections. Generally, a cocktail of distinct phages provides robust protection, due to various available phage receptors, and long-lasting protection due to limited possibilities of resistance development (Gill and Hyman, 2010). Phage therapy is still in its infancy as far as the treatment of crop infections is concerned. However, there are many examples of bacteriophages being isolated against plant pathogenic bacteria of crops, and phage cocktail applications are gradually becoming more common (Doss et al., 2017 and references therein). Even though the mechanism of bacteriophage cocktails could fit in either category of the control strategies described in this review, its therapeutic character is emphasized here.

In the case of XYLEFA, a temperate phage was induced from a *Xff* strain supernatant (Summer et al., 2010). Phage therapy applies only virulent phages since temperate phages are unstable and could incorporate into the host genome, accidentally transfer genes (transduction) and/or confer superinfection exclusion (Gill and Hyman, 2010). The first virulent phages against XYLEFA have recently been described by Ahern et al. (2014). The group isolated four broad-host range phages, i.e., phages that could infect many different strains of XYLEFA and some strains of *Xanthomonas* species. Two of the isolated phages, Sano and Salvo, belong to the family of *Siphoviridae*, while the other two, Prado and Paz, belong to the family of *Podoviridae* (Ahern et al., 2014). Grapevines were inoculated, either pre or post-*Xff* infection, with a cocktail of all four phages, using a titer of 1 × 10^10^ PFU/ml. In both experiments, the phage cocktail was able to fully protect vines against *Xff* infection in greenhouse experiments (Das et al., 2015). In another greenhouse-based study, GWSS were used as vectors of phage Paz. Even though GWSS were able to efficiently acquire phage Paz, they could not transmit it to cowpea plants (Bhowmick et al., 2016). All in all, phage therapy is a promising field and more studies are needed to investigate the potential of phages for treating PD and plant bacterial infections in general.

### Antagonistic bacterium *paraburkholderia phytofirmans* strain PsJN

The use of biocontrol bacteria against *Xff* has been presented in the “preventive strategies” section. Nevertheless, *Paraburkholderia phytofirmans* strain PsJN is also therapeutic since it has been quite successful in curing infected vines. PsJN was first isolated from onion roots (Sessitsch et al., 2005) and successfully colonized vines epiphytically and endophytically during in planta trials (Compant et al., 2008). Although the titer and spread of PsJN within the vines 6 weeks after inoculation resembled that of XYLEFA, population sizes measured 12 weeks after inoculation were quite low (Lindow et al., 2015). *In planta* experiments demonstrated that the endophyte can inhibit growth of XYLEFA in various vine cultivars when co-inoculated or even when inoculated 30 days after the pathogen (Lindow et al., 2017a). Another attractive characteristic is that PsJN can control PD not only when inoculated but also when applied foliarly, provided it is combined with a penetrative surfactant (Lindow et al., 2015). Field experiments and further attempts to decipher how PsJN mediates control of PD are in progress (Lindow et al., 2017a). Results indicate that PD mitigation does not derive from a putative *rpfF* gene of PsJN. Instead, a small molecule, active in low concentrations may interfere with *Xff* quorum-sensing regulation and biofilm formation (Lindow et al., 2017a). Furthermore, it is hypothesized that, together with the signaling molecule, PsJN may trigger host immune defense responses (Lindow et al., 2017a).

### Natural, antibacterial substances

Up to now, the application of chemical compounds has played a leading role in agriculture and been a widespread practice for controlling plant pathogens in the field. However, recent legislation, such as Directive 2009/128/EC (European Parliament Council, 2009), requires the reduction of synthetic pesticides in agriculture owing to their environmental impact and risks to human health (Dayan et al., 2009). Thus, there is a particular requirement to discover and develop more natural pesticides to replace their chemical counterparts (Dayan et al., 2009). Fortunately, the alternative of applying natural product-based compounds is not wholly new to agricultural scientists.

In the case of PD, researchers have tried to identify and evaluate various natural, antibacterial substances against *Xff*. One such study assessed the use of fungal natural products that can inhibit XYLEFA *in vitro* (Rolshausen and Roper, 2016). First, radicinin produced by *Cochliobolus* sp., and later crude extracts from the endophytes *Eurotium* sp., *Geomyces* sp., and *Ulocladium* sp. successfully inhibited XYLEFA *in vitro* (Rolshausen et al., 2015). Radicinin is a known, naturally produced fungal compound, and its antibacterial and phytotoxic properties are well described (Aldrich et al., 2015 and references therein). Radicinin's mode of action against XYLEFA is *via* a mechanism that involves inactivation of *Xff* proteases (Aldrich et al., 2015). Radicinin is currently being tested for its curing potential *in planta* under greenhouse conditions (Rolshausen et al., 2017).

In another study on antibacterial molecules, (Kuzina et al., 2006) screened PD and other XYLEFA strains using various antibiotics and antimicrobial peptides. Earlier, Kirkpatrick et al. had tested the antibiotic tetracycline *in vitro* (Kirkpatrick et al., 2001) and the antibiotic streptomycin *in planta* (Kirkpatrick et al., 2004) and found them to be effective against XYLEFA. The study of those antibiotics was moved to the field and a report on their effect is still pending. The study of Kuzina et al. was restricted to *in vitro* assays, but is still noteworthy due to the extent of the antibacterial molecules tested. Of the 12 antibiotics tested, the best candidates for XYLEFA control were gentamicin (0.5–1 μg/ml), tetracycline (1–4 μg/ml), ampicillin, kanamycin, and novobiocin (4–8 μg/ml), chloramphenicol (1–8 μg/ml), and rifampin (2–8 μg/ml) (ibid). Moreover, out of the 18 antimicrobial peptides tested, only four, namely PGQ, indolicidin, magainin 2, and dermaseptin, strongly inhibited all XYLEFA strains in the study (Kuzina et al., 2006). On the one hand, further studies are necessary to develop the right delivery system of those molecules to grapevines (Kuzina et al., 2006). On the other hand, it is less likely that any antibacterial molecules will be permitted for in planta applications against *Xff*, because of their adverse killing of other plant beneficial microbes and their use in human therapy.

A similar study investigated the anti-Xylella effect of 12 phenolic compounds *in vitro* (Maddox et al., 2010). The researchers studied two PD strains and two strains infecting almond for their sensitivity to caffeic acid, catechin, p-coumaric acid, resveratrol, rutin, sinapic acid, catechol, coumarin, ferulic acid, gallic acid, naringenin, and quercetin (Maddox et al., 2010). All the tested compounds were able to efficiently inhibit *Xff*, but catechol, caffeic acid and resveratrol had the greatest effect (Maddox et al., 2010). Nonetheless, the mechanism of inhibition of those compounds remains unknown. Lastly, potted PD-infected vines were inoculated with the microelement zinc sulfate/oxide (Kirkpatrick et al., 2004). The vines were assessed over a three-year period and exhibited noteworthy recovery from PD (Kirkpatrick et al., 2004). Even though no results of field studies have been published yet, the use of phenolics may be restricted, owing to their wide range of inhibition that can also impact beneficial microbes of vines.

Other molecules with antibacterial properties have been tested *in planta* and produced promising results. Nevertheless, the host challenged was not grapevine so they will only be mentioned briefly here. N-Acetylcysteine, a molecule used in medicine to disrupt disulfide bonds in mucus, was able to reduce citrus variegated chlorosis symptoms *in planta* (Muranaka et al., 2013). In another study an antimicrobial peptide from a tarantula spider, gomesin, interfered with two citrus variegated chlorosis strains and reduced their virulence *in planta* (Fogaça et al., 2010). Generally, the combination of molecules that can reduce biofilm formation of XYLEFA with antimicrobial compounds is of good potential and should be considered in future. This is significant considering that biofilms greatly increase bacterial resistance to antimicrobial compounds (Mah and O'Toole, 2001). On the other hand, strategies of biofilm reduction can be efficient during the early stages of XYLEFA infection. In later stages, reduction of virulence has been attempted following the opposite approach, i.e., increased adhesiveness and reduced motility, such as in the study of Lindow et al. (2014).

### Other defense-stimulating compounds

Defense-stimulating compounds have also been included in trials against *Xff*. The first case study, by Meyer and Kirkpatrick (2011), investigated the curative effect of ABA. This is a typical plant hormone that regulates plant growth, seed dormancy, and stomatal function. In addition to these roles, ABA is an important factor in plant responses to a disease. In a greenhouse trial, Meyer and Kirkpatrick (2011) applied ABA to grapevines infected by *Xff via* foliar sprays or soil drenches. The trial ran for three consecutive years and the exogenous ABA applications were performed using one naturally occurring and one synthetic ABA (Meyer and Kirkpatrick, 2011). The treated vines exhibited significant recovery from PD, and the researchers further noted a significant rise in the phenolic compound concentration in those vines. This implied a positive correlation between successful ABA treatments and the content of phenolic compounds in the sap (Meyer and Kirkpatrick, 2011). Other than ABA, metal chelators have been recommended for the inhibition of *Xff*. Koh and Toney (2005) reported that biofilm formation can be blocked *via* iron chelators such as lactoferrin, EDTA (ethylenediaminetetraacetic acid) and EDDS (ethylenediamine-N, N′-disuccinic acid). It was hypothesized that the possible mechanism of action is connected to iron deprivation for XYLEFA (Koh and Toney, 2005).

This review concerns control strategies already tested but many more studies could serve as a source of inspiration for the development of efficient strategies against *Xff*. For example, a recent study by Zaini et al. (2018) has specified various response mechanisms of vines to *Xff* infection that could be exploited, including accumulation of gamma-aminobutyric acid and upregulation of phytoalexins. As presented in this first section, various prophylactic and therapeutic strategies have already been proposed in order to minimize the effects of *Xff* in vineyards. A substantial number of these strategies appear promising. To evaluate the efficacy of all current and future approaches, the following section will model the spread of *Xff* in a population of vines.

## *Xff* epidemiological model

### Description of the *Xff* epidemiological model

This section elaborates an epidemiological model of *Xff* with the aim of deciphering key parameters for the spread of PD. To the authors' knowledge, only one relevant model has so far been published (Perring et al., 2003). Given that the model of Perring et al. (2003) is still in a preliminary form, the *Xff* epidemiological model made in the following section was based on a malaria model by Chitnis (2005). To calibrate the present model, SC was used as a reference due to the consistency of studies and the severity of PD there (Rapicavoli et al., 2017). Even though the model presented here is expected to be applicable to any high-transmission region of *Xff*, it would be advisable to perform these analyses again with suitable parameter values. This will allow users to verify whether the significance of various parameters remains as reported here for the new regions of interest.

#### Definition of the vine state variables of the *Xff* epidemiological model

**Figure 2:**
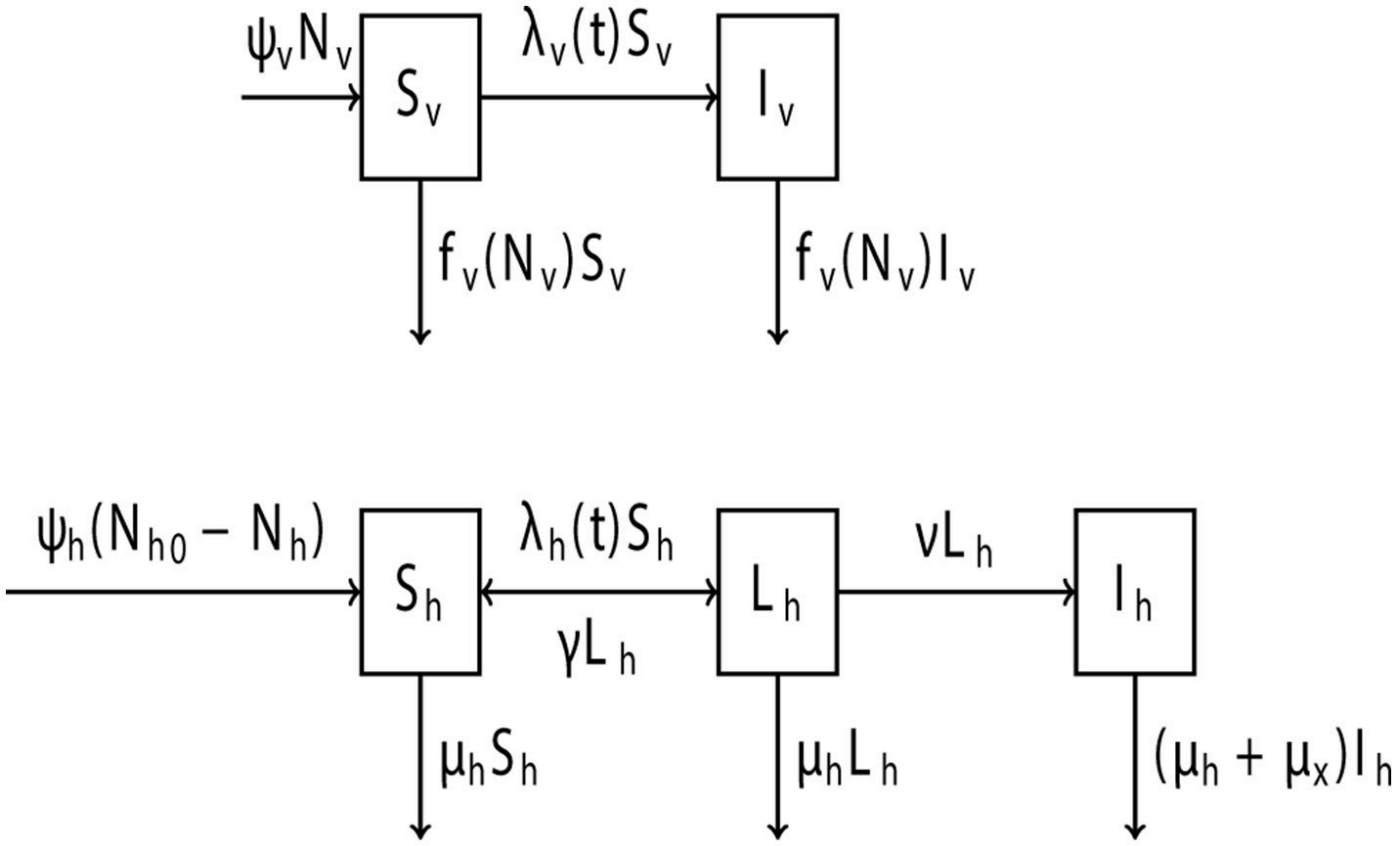
Schematic of the *Xff* epidemiological model showing the transition of the vine and GWSS populations to different stages due to *Xff* presence. State variables referring to the stages of GWSS are healthy *S*_*v*_ and infected *I*_*v*_. State variables referring to the stages of vines are healthy *S*_*h*_, latent *L*_*h*_ and symptomatic *I*_*h*_. Displayed on each transition between the state variables are the rates at which they happen in the model (see Equations 1–5). The model parameters can be found in Table 4.

This model describes the dynamics of a vine population under high PD pressure. The *Xff* epidemiological model (Figure 2) divides the vine population into three classes: healthy (S_h_), latent (L_h_), and symptomatic (I_h_). A vine becomes latent only after an infected GWSS vector manages to probe the vine and successfully inject *Xff* into the vine's xylem (Killiny and Almeida, 2014). Generally, a probe involves all GWSS behaviors starting from the penetration of its stylet and ending with stylet withdrawal (Sandanayaka and Backus, 2008). Once in the latent stage, a vine does not yet display any PD symptoms, but harbors sufficient *Xff* titers to infect a healthy GWSS (Hill and Purcell, 1997; Schaad et al., 2002). Latent vines can then either recover or start developing symptoms. Vines that enter the latent stage in late summer often recover and revert to the healthy stage if the following winter is cold. Low winter temperatures are hostile to *Xff* growth and do not allow the pathogen to become established inside the vine (Feil and Purcell, 2001; Purcell, 2013). Furthermore, severe winter pruning may enhance, but not replace, the curing effect of low temperatures (Daugherty et al., 2018). Vines that entered the latent stage during early spring and summer become symptomatic within two to 12 months (Hopkins and Purcell, 2002). Symptomatic vines cannot recover and will decline within a year of infection at the earliest (Varela et al., 2015).

#### Definition of the GWSS state variables of the *Xff* epidemiological model

As experience in the case of SC, GWSS is the insect vector likely to lead a vine population to a PD epidemic (Rapicavoli et al., 2017). For the *Xff* model, the GWSS population was divided into two classes: healthy (S_v_) and infected (I_v_). A healthy GWSS would pass into the infected stage only after successfully probing a vine that is either latent or symptomatic. Generally, plants other than vine can also infect GWSS (Almeida et al., 2005a; Janse and Obradovic, 2010). However, such plant hosts are either non-existent or inconsiderable sources of infection in SC (Mizell et al., 2008a; Almeida and Nunney, 2015), the region used in this model as a reference. In GWSS, *Xff* multiplies in the vector's foregut (Purcell et al., 1979) and can be transmitted by that GWSS in less than an hour (Almeida and Purcell, 2003). GWSS has neither transovarial nor transstadial transmission of *Xff*, but nymphs of the vector may carry *Xff*, and adults that acquire *Xff* remain infected for life (Almeida et al., 2005a; Chatterjee et al., 2008). New GWSS are born at a constant per capita birth rate. For the *Xff* model, it was assumed that GWSS are “born” as adults. This is a simplifying but realistic assumption, since the adult stage lasts substantially longer and is generally more active in the dispersal of *Xff* than any nymphal stage of GWSS (Mizell et al., 2008a; Lauzière and Sétamou, 2010). The acquisition of *Xff* appears to have no effect on GWSS longevity or fertility (Mizell et al., 2008b).

#### Differential equations of the *Xff* epidemiological model

To model the spread of *Xff* in a population of vines, the following system of differential equations was used:

(1) $$\begin{array}{l} \frac{dS_{v}}{dt}=\psi_{v}N_{v}-\lambda_{v}\left(t\right)S_{v}-f_{v}\left(N_{v}\right)S_{v} \end{array}$$

(2) $$\begin{array}{l} \frac{dI_{v}}{dt}=\lambda_{v}\left(t\right)S_{v}-f_{v}\left(N_{v}\right)I_{v} \end{array}$$

(3) $$\begin{array}{l} \frac{dS_{h}}{dt}=\psi_{h}\left(N_{h0}-N_{h}\right)-\lambda_{h}\left(t\right)S_{h}+\gamma L_{h}-\mu_{h}S_{h} \end{array}$$

(4) $$\begin{array}{l} \frac{dL_{h}}{dt}=\lambda_{h}\left(t\right)S_{h}-\gamma L_{h}-\nu L_{h}-\mu_{h}L_{h} \end{array}$$

(5) $$\begin{array}{l} \frac{dI_{h}}{dt}=\nu L_{h}-\left(\mu_{h}+\mu_{x}\right)I_{h} \end{array}$$

The state variables of the model are shown in Table 3 and the parameters used in the model are shown in Table 4.

**Table 3:** State variables of the *Xff* epidemiological model.

*N_*v*_*	Total number of GWSS
*S_*v*_*	Number of healthy GWSS
*I_*v*_*	Number of infected GWSS
*N_*h*_*	Total number of vines
*S_*h*_*	Number of healthy vines
*L_*h*_*	Number of latent vines
*I_*h*_*	Number of symptomatic vines

**Table 4:** Parameters of the *Xff* epidemiological model.

*ψ_*v*_*	Per capita birth rate of GWSS, time ^−1^
*ψ_*h*_*	Per capita replacement rate of (missing) vines, time ^−1^
*N_*h*0_*	The maximum number of vines, unit
*μ_*v*1_*	Density-independent part of GWSS death rate, time ^−1^
*μ_*v*2_*	Density-dependent part of GWSS death rate, vector ^−1^× time ^−1^
*f*_*v*_(*N*_*v*_) = μ_*v*1_+ μ_*v*2_*N*_*v*_	Per capita density-dependent death rate of GWSS, time ^−1^
*μ_*h*_*	PD-independent death and removal rate of vines, time ^−1^
*μ_*x*_*	PD-induced removal rate of vines, time ^−1^
$\lambda_{v}\left(t\right)=\frac{\beta_{hv}p\sigma \left(L_{h}+\;I_{h}\right)}{N_{h}}$	Per capita inoculation rate for GWSS, time ^−1^
$\lambda_{h}\left(t\right)=\frac{\beta_{vh}p\sigma I_{v}}{N_{h}}$	Per capita inoculation rate for vines, time ^−1^
*β_*hv*_*	Probability of transmission from vine to GWSS during a probe, dimensionless
*β_*vh*_*	Probability of transmission from GWSS to vine during a probe, dimensionless
σ	Number of probes a GWSS performs on vines per unit of time, dimensionless
ν	Rate of progression from latent to symptomatic vine, time ^−1^
γ	Rate of recovery for latent vines, time ^−1^

#### Expression of the differential equations in the fractional system

To facilitate analysis, state variables over the total populations were expressed as

$$\begin{array}{l} i_{v}=\frac{I_{v}}{N_{v}};\;s_{v}=1-i_{v};\;s_{h}=\frac{S_{h}}{N_{h0}};\;l_{h}=\frac{L_{h}}{N_{h0}};\;i_{h}=\frac{I_{h}}{N_{h0}} \end{array}$$

in order to arrive at an equivalent system in terms of fractional quantities

(6) $$\begin{array}{l} \frac{di_{v}}{dt}=\lambda_{v}\left(t\right)\left(1-i_{v}\right)-\psi_{v}i_{v} \end{array}$$

(7) $$\begin{array}{l} \frac{dN_{v}}{dt}=\psi_{v}N_{v}-f_{v}\left(N_{v}\right)N_{v} \end{array}$$

(8) $$\begin{array}{l} \frac{ds_{h}}{dt}=\psi_{h}\left(1-s_{h}-l_{h}-i_{h}\right)-\lambda_{h}\left(t\right)s_{h}+\gamma l_{h}-\mu_{h}s_{h} \end{array}$$

(9) $$\begin{array}{l} \frac{dl_{h}}{dt}=\lambda_{h}\left(t\right)s_{h}-\gamma l_{h}-vl_{h}-\mu_{h}l_{h} \end{array}$$

(10) $$\begin{array}{l} \frac{di_{h}}{dt}=vl_{h}-\left(\mu_{h}+\mu_{x}\right)i_{h} \end{array}$$

### Baseline parameter values

As mentioned earlier, baseline parameter values were chosen that refer to SC. These values are presented below (Table 5) and an explanation given for their selection. All the values utilized are estimates from the literature.

**Table 5:** Baseline parameter values applied to calibrate the *Xff* epidemiological model.

*ψ_*v*_*	0.32, 2.1 eggs per female per day, 30% of which survive
*ψ_*h*_*	1/365, giving an average replacement time of 365 days
*N_*h*0_*	10000
*μ_*v*1_*	0.01, giving an average lifetime of 100 days
*μ_*v*2_*	1.55 × 10^−5^, which leads to Nv*Nh0=2
*μ_*h*_*	1.1 × 10^−4^, giving an average lifetime of 25 years
*μ_*x*_*	1/180, giving an expected time of vine removal once symptomatic of 180 days
*β_*hv*_*	0.2
*β_*vh*_*	0.35
σ	1.5, GWSS performs 5 probes, 30% of which are on vines
ν	1/120, average time of progressing 120 days
γ	0.0033, giving a 28% chance of recovery once a vine has become latent

#### Model parameters for GWSS

Since vines contract PD only *via* specialized insect vectors, parameters that describe GWSS behavior are essential to this model. Only the adult stage of the GWSS life cycle was included, thus the birth rate was adjusted to account for egg and nymphal survival. One female GWSS lays 2.1 eggs per day on average during the active oviposition period (Sétamou and Jones, 2005). It was assumed that eggs are produced through a constant per capita birth rate, and that 50% of all female GWSS lay eggs every day (Sisterson, 2008). Based on the values given by Lauzière and Sétamou (2010), it was estimated that 30% of the total number of eggs would survive due to environmental pressures, such as egg parasitism, rather than greenhouse conditions. Therefore the per capita birth rate, ψ_*v*_, of GWSS was 0.32 per day and the per capita density-dependent death rate of GWSS was (μ_*v*1_ + μ_*v*2_ × *N*_*v*_).

**Figure 3:**
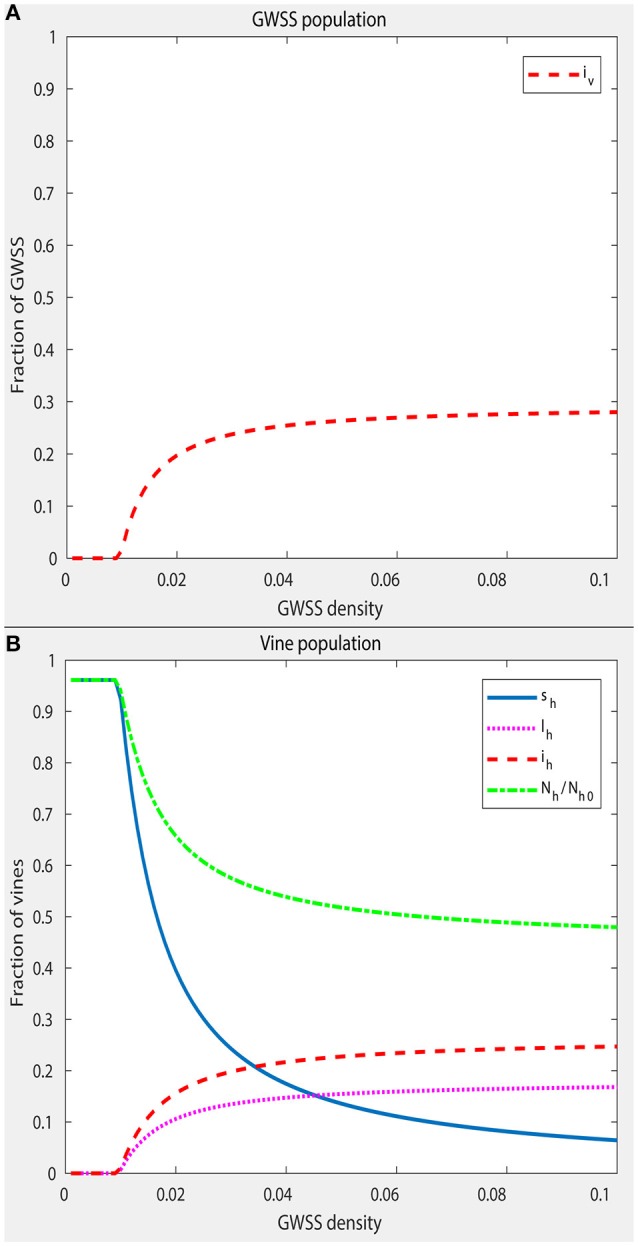
Equilibrium prevalence of PD in the GWSS population **(A)** and in the vine population **(B)**, as a function of GWSS density, i.e., the relation between the equilibrium total number of GWSS, N^*^and the maximum number of vines *N*_*h*0_. The equilibrium values are presented using the expression of the state variables in the fractional system. These are infected GWSS over the total number of GWSS *i*_*v*_, healthy vines over the maximum number of vines *s*_*h*_, latent vines over the maximum number of vines *l*_*h*_ and infected vines over the maximum number of vines *i*_*h*_. Denoted as *N*_*h*_/*N*_*h*0_ is the number of vines in the population at a given time over the maximum number of vines. To obtain the equilibria at different GWSS densities, a numerical simulation was run with each value, starting from an initial low level of infection (*i*_*v*_ = 0.1 and *s*_*h*_ = 1) until the system had converged to a steady state. Other parameter values remain as in Table 5. The plot shows that PD becomes endemic at a GWSS density of ~0.01 GWSS per vine.

**Figure 4:**
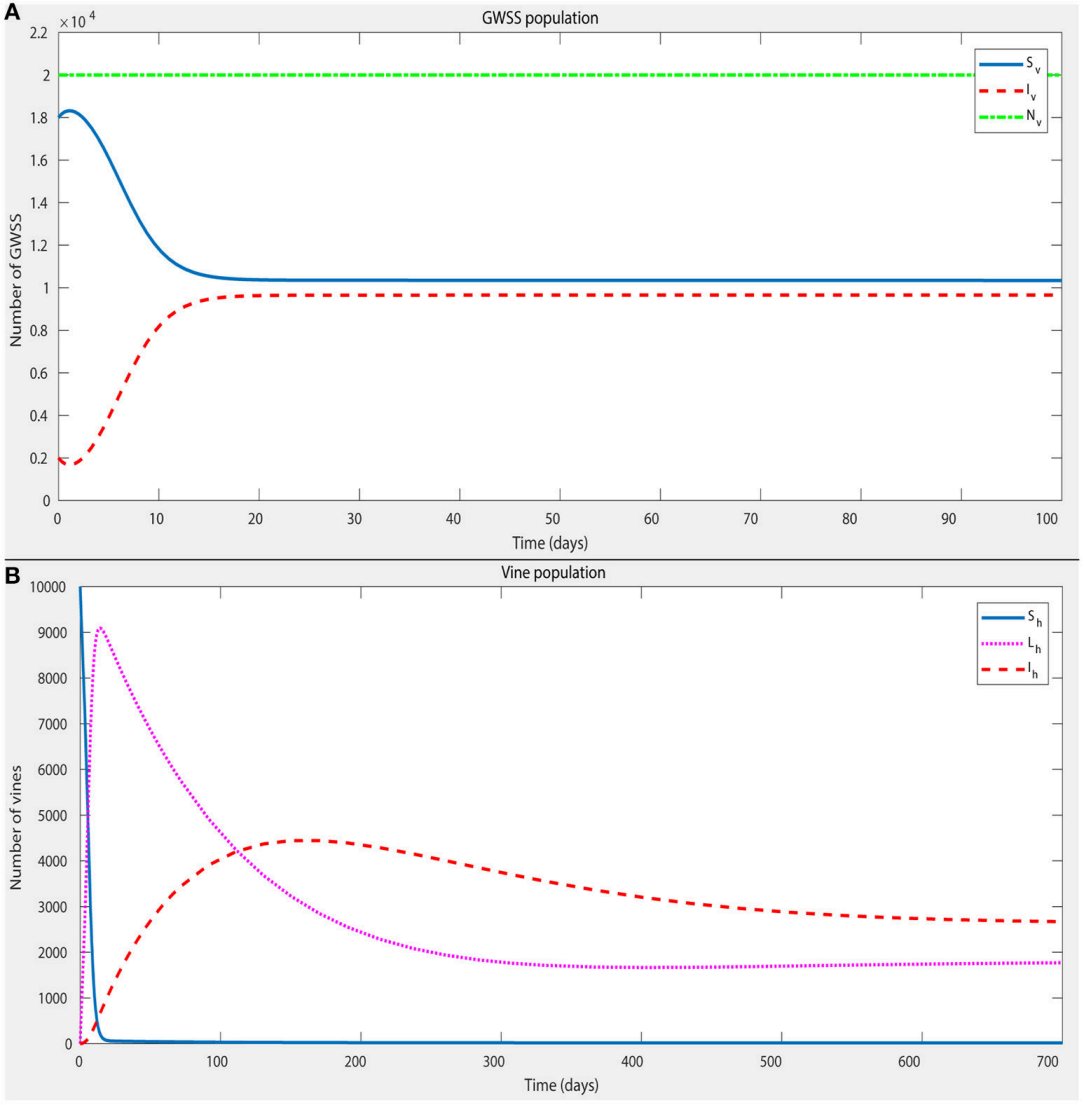
A numerical simulation of the model, with parameter values given in Table 5, in order to confirm the existence of a stable endemic equilibrium. The initial conditions for the state variables (Table 3) were: *S*_*h*_ = 10,000; *L*_*h*_ = 0; *I*_*v*_ = 0; *S*_*v*_ = 18,000; *I*_*v*_ = 2,000. The plot shows that a stable endemic equilibrium is indeed established within ~2 years for both the GWSS and vine populations (**A,B**, respectively).

The density-independent part of the death rate of GWSS, μ_*v*1_, was set at 1/100 per day, which is in agreement with the already reported longevity of this vector (Lauzière and Sétamou, 2010). The density-dependent part, μ_*v*2_, is the parameter that largely controls the equilibrium size of the GWSS population. For most of this analysis, a GWSS population of approximately twice the size of the vine population was used, in conditions when PD is not present. This estimate was based on Krugner et al. (2012), the 2016 Citrus Acreage Report (NASS/CDFA, 2017a) and the 2016 Grape Acreage Report (NASS/CDFA, 2017b). To obtain such a size of GWSS population, μ_*v*2_ was set accordingly. However, μ_*v*2_ was also varied to simulate situations with different numbers of GWSS per vine (spatial distribution of vectors) and to explore how this affects the disease dynamics (Figures 3, 4).

The probability of transmission from vine to vector during a probe, β_*hv*_, is known as vector acquisition efficiency in the XYLEFA vector literature, and the probability of transmission from vector to vine during a probe, β_*vh*_, as vector inoculation efficiency. GWSS acquisition efficiency is poor when the titer of *Xff* in the vine is low and can increase when probing duration increases, but only for a period of up to 6 h (Almeida and Purcell, 2003 and references therein). GWSS inoculation efficiency for a GWSS individual depends on probing duration (Almeida and Purcell, 2003). Nevertheless, the existence of other factors affecting GWSS inoculation and acquisition efficiency should be clarified in order to design successful control strategies (Daugherty and Almeida, 2009). Both probability values were extracted from the literature (Almeida and Purcell, 2003).

The number of probes on vines were set per unit of time at σ = 1.5, after combining information on the number of probes (Sandanayaka and Backus, 2008) and GWSS preference for vine (Daane et al., 2003; Mizell et al., 2008a). Moreover, the impact of seasonality and field conditions were also incorporated because they reduce the number of probes a GWSS can perform on a host plant (Blua and Morgan, 2003). It should be noted that this definition of σ describes a fairly local situation, where it is assumed that the total number of probes the GWSS population performs is distributed evenly across all vines. Therefore, this model does not account for vector aggregation and swarm behavior, although it is acknowledged that these factors can significantly influence disease spread. This concept also affects the per capita rate of inoculation, λ, which is a function of the number of probes on vines, the proportion of infected vectors, and the probability of transmission per probe.

#### Model parameters for vines

The vine population is an artificial one, and its maximum size *N*_*h*0_ considered to be the number that the vine-grower aims to maintain. Vines are being removed at a constant rate, μ_*h*_, because of age and other diseases. The PD-independent death and removal rate of vines, was set at 1.1 × 10^−4^ per day to reach an average lifespan of 25 years. This choice is in accordance with practices of Californian vineyards (Alston et al., 2013). In SC, vine-growers are advised to rogue vines within the first year of PD symptom development (Varela et al., 2015). Vines start showing symptoms within two to 12 months of infection (Hopkins and Purcell, 2002). Considering these two factors, the PD-induced death and removal rate of vines, μ_*x*_, was estimated to be equal to 1/180 per day, so that the average time it takes for a symptomatic vine to be removed is 6 months. The missing vines, corresponding to the difference between *N*_*h*0_ and *N*_*h*_, are being replaced at a constant per capita rate ψ_*h*_. Consequently, in the absence of PD, vines have a stable population level *N*_*h*_ < *N*_*h*0_.

The per capita replacement of missing vines, ψ_*h*_, was set at 1/365 per day to reflect viticulture guidelines and nurseries' responsiveness (Alston et al., 2013; Daniels et al., 2017). It was assumed that all vines that enter the population from a nursery are free of *Xff*. Latent vines recover at a constant rate, γ, or progress to the symptomatic stage at a constant rate, ν. It was gauged that the cold-curing effect and severe winter pruning support a 28% chance of recovery (Lieth et al., 2011; Daugherty et al., 2018), thus γ = 0.0033 per day. Alternatively, latent vines become symptomatic in an average of 120 days (Li et al., 2002), thus the progression from latent to symptomatic vine, ν, was estimated to be 1/120 per day.

### Disease-free equilibria

The dynamics of the total number of GWSS *N*_*v*_ is not affected by the disease. Hence, the equilibrium number of GWSS is always given by $\frac{dN_{v}}{dt}=0↔N_{v}=0$ or $N_{v}=\frac{\psi_{v}-\mu_{v1}}{\mu_{v2}}$. Clearly, the non-trivial equilibrium exists only if ψ_*v*_ > μ_*v*1_, and is asymptotically stable. It is denoted by $N_{v}^{*}$.

For the vine, in the absence of disease the only dynamics are:

$$\frac{ds_{h}}{dt}=\psi_{h}\left(1-s_{h}\right)-\mu_{h}s_{h},$$

that is the non-PD-related death and removal, and subsequent replacement. This gives the disease-free equilibrium for vine $s_{h}^{*}=\frac{\psi_{h}}{\psi_{h}+\;\mu_{h}}$, which equals approximately 0.96 for the parameter values in Table 5. Taken together, the disease-free equilibrium of the whole system is $i_{v},N_{v},s_{h},l_{h},i_{h}=\left(0,N_{v}^{*},s_{h}^{*},0,0\right)$.

### Basic reproduction number

The basic reproduction number *R*_0_ is a metric to determine whether an infection will spread in a healthy population. There are different definitions and ways to calculate it. Generally, though, *R*_0_ aims to approximate the number of secondary infections one infected individual will cause when it enters a fully susceptible population. Thus *R*_0_ < 1 would mean the disease will not establish itself. Following Diekmann et al. (2009), the basic reproduction number was calculated using

$$R_{0}=\sqrt{K_{vh}K_{hv}}$$

where *K*_*vh*_ and *K*_*hv*_ are, respectively, the number of vines one infected GWSS is expected to infect and the number of GWSS to which one infected vine is expected to transmit the pathogen, assuming a completely healthy population. *K*_*vh*_ and *K*_*hv*_ are given by

$$\begin{array}{l} K_{vh}=\frac{\sigma \beta_{vh}}{\mu_{v1}+\mu_{v2}N_{v}^{*}}\\ K_{hv}=\frac{\sigma \beta_{hv}N_{v}^{*}}{s_{h}^{*}N_{h0}}\frac{1}{\gamma +v+\mu_{h}}+\frac{v}{\gamma +v+\mu_{h}}\frac{\sigma \beta_{hv}N_{v}^{*}}{s_{h}^{*}N_{h0}}\frac{1}{\mu_{h}+\mu_{x}}. \end{array}$$

**Figure 5:**
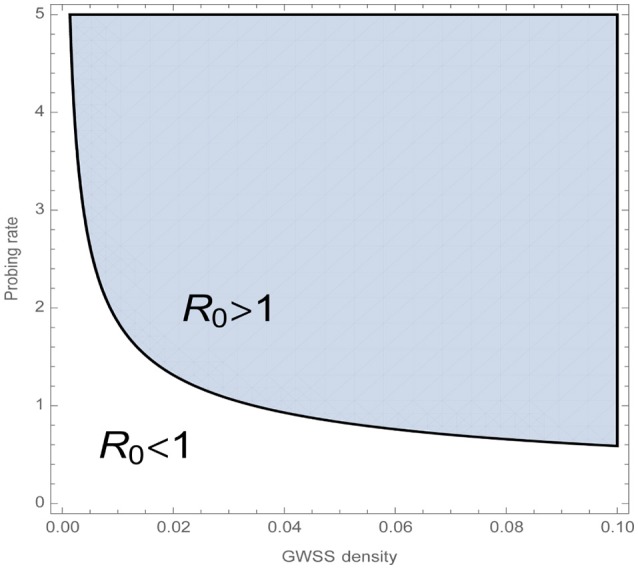
The basic reproduction number *R*_0_ indicates whether a disease can establish itself in a healthy population through an initial infection. Namely when *R*_0_ < 1, the disease-free equilibrium of the whole system, $i_{v},\;N_{v},\;s_{h},\;l_{h},\;i_{h}=\left(0,N_{v}^{*},s_{h}^{*},\;0,\;0\right),$ is stable and a small number of infected individuals will not cause endemic PD. GWSS density is defined as $N^{*}/N_{h0}$, which is the relation between the equilibrium total number of GWSS, N^*^, and the maximum number of vines *N*_*h*0_. The plot shows how the combined increase in GWSS density and probing rate positively affects *R*_0_. The rest of the model parameters are fixed to the values given in Table 5.

*K*_*vh*_ translates as the product of GWSS expected lifetime and the rate at which the vector successfully inoculates a vine. *K*_*hv*_ equals the expected time the vine spends at the latent stage multiplied by the number of successful transmissions to a GWSS. The second summand of *K*_*hv*_ describes the same, but for the symptomatic stage, multiplied by the probability of transitioning from the latent to the symptomatic stage. Figure 5 displays how the basic reproduction number depends on the probing rate, σ, and GWSS density, i.e., the relation between the equilibrium total number of GWSS, N^*^and the maximum number of vines*N*_*h*0_. Specifically, it shows that a combined increase in GWSS density and probing rate positively affects the chances of establishment of endemic PD in the vine population.

### Endemic equilibrium

An endemic equilibrium is a steady state solution in which the disease will persist in the system. Numerical simulations of this system indicate that there is a unique endemic equilibrium and that it is asymptotically stable when *R*_0_ > 1. To examine how GWSS density affects the equilibria, simulations were performed for different values of GWSS density. For each value of vector density, a numerical simulation was started with a small initial number of infected individuals (*i*_*v*_ = 0.1 and *s*_*h*_ = 1) and run until sufficient convergence was achieved. The obtained results are displayed in Figure 3 and show that PD becomes endemic at a GWSS density of approximately 0.01 GWSS per vine (Figures 3A,B).

To show that this system indeed reaches an endemic equilibrium for the baseline parameter values of Table 5, a numerical simulation was run starting from a low initial *Xff* presence in the system (*S*_*h*_ = 10,000; *L*_*h*_ = 0; *I*_*v*_ = 0; *S*_*v*_ = 18,000; *I*_*v*_ = 2,000). The simulation yielded persistence of *Xff* in the system and establishment of a stable endemic equilibrium within approximately two years for both the GWSS and vine populations (Figures 4A,B, respectively).

Unfortunately, the aforementioned equilibria could not be solved analytically. Rigorous proof of their existence and type falls outside the scope of this paper.

### Sensitivity analysis and identification of key parameters for PD control

The aim of this model was to assess the importance of different parameters of PD epidemiology and, through them, suggest which control strategies would be more efficient against *Xff* spread. To that end, a sensitivity analysis was performed for the model parameters following the approach detailed in Arriola and Hyman (2009). The normalized sensitivity index of output *u* to parameter *p* is defined as

(11) $$\begin{array}{l} S_{up}=\frac{p}{u}\frac{\partial u}{\partial p} \end{array}$$

This estimates how the output *u* changes when small perturbations are made to the value of *p*. By calculating the sensitivity index for the basic reproduction number, *R*_0_, it could be determined which parameters of the model are responsible for the initial transmission of *Xff*. Meanwhile, the sensitivity index was also calculated for the endemic equilibrium and therefore key parameters that induce PD prevalence pinpointed. Given the analytical expression for *R*_0_, its sensitivity to the 12 parameters of the *Xff* model could be directly computed. For the endemic equilibrium, a linear system for each parameter was solved first in order to find an expression for *S*_*up*_. This expression was then evaluated at the numerically solved equilibrium value of the state variables. The results of the sensitivity analysis for the different parameters of this model are presented in Tables 6, 7.

**Table 6:** Sensitivity indices of the basic reproduction number *R*_0_ to the parameters, evaluated at the values given in Table 5.

***R***_**0**_	
*ψ_*v*_*	0.016
*ψ_*h*_*	−0.019
*N_*h*0_*	−0.5
*μ_*v*1_*	−0.016
*μ_*v*2_*	−0.5
*μ_*h*_*	0.0088
*μ_*x*_*	−0.29
*β_*hv*_*	0.5
*β_*vh*_*	0.5
σ	1
ν	−0.057
γ	−0.14

**Table 7:** Sensitivity indices of the endemic equilibrium values of the state variables to the parameters, evaluated at the values given in Table 5.

	***i_*v*_***	***N_*v*_***	***s_*h*_***	***l_*h*_***	***i_*h*_***
*ψ_*v*_*	−0.77	1.0	−0.27	0.00079	0.0023
*ψ_*h*_*	0.7	0.0	0.76	0.55	1.6
*N_*h*0_*	−0.0038	0.0	1.0	−0.003	−0.0088
*μ_*v*1_*	−0.00012	−0.032	0.032	−0.000096	−0.00028
*μ_*v*2_*	−0.0038	−1.0	1.0	−0.003	−0.0088
*μ_*h*_*	−0.012	0.0	0.00025	−0.0056	−0.036
*μ_*x*_*	−0.014	0.0	0.048	0.088	−0.72
*β_*hv*_*	0.77	0.0	−0.77	0.0023	0.0067
*β_*vh*_*	0.0038	0.0	−1.0	0.0030	0.0088
σ	0.77	0.0	−1.8	0.0052	0.016
ν	−0.27	0.0	−0.18	−0.81	0.56
γ	−0.0011	0.0	0.28	−0.00083	−0.0025

The most sensitive parameter for *R*_0_ is the number of probes a GWSS performs on a vine per unit of time, σ. The sign of this sensitivity index means that as *S*_*R*0σ_ = 1, decreasing (or increasing) σ by 10% would decrease (or increase) *R*_0_ by 10%. In other words, the initial transmission of *Xff* would decrease (or increase) by 10%. Other noteworthy parameters are the probability of transmission from vine to GWSS during a probe, β_*hv*_, and the probability of transmission from GWSS to a vine during a probe, β_*vh*_. Similar to σ, these two parameters are positively correlated to *R*_0_. Finally, the maximum number of vines, *N*_*h*0_, and the density-dependent part of GWSS death rate, μ_2*v*_, significantly affect *R*_0_, and are negatively correlated to it. For most of the parameters, the sign of the sensitivity index of *R*_0_ agrees with an intuitive expectation. Exceptions are the rate of progression from latent to symptomatic, ν, the maximum number of vines, *N*_*h*0_, and the PD-independent death and removal rate of vines, μ_*h*_.

According to these results (Table 6), the reproduction number increases when the rate of progression from latent to symptomatic decreases. This is because latent vines are a source of infection that cannot be traced and removed by the vine-grower. Thus, the existence of more latent vines in a population would mean more frequent encounters with *Xff* for GWSS. The reproduction number also increases when the maximum number of vines decreases. This is interpreted as being due to a shrinkage of probing options for GWSS. As the maximum number of vines drops, the chances rise that healthy GWSS would end up probing on vines with PD and successfully acquiring the pathogen. Furthermore, the chances that infected GWSS feed on a healthy vine and successfully inoculate *Xff* also rise, causing an overall increase in transmission. Lastly, the reproduction number increases as the PD-independent death and removal rate increases. This happens because the vine population becomes smaller, which enhances the possibility that GWSS would feed on a vine with PD and acquire the pathogen. However, the increase in death and removal also decreases the lifetime of a vine, which has the opposite effect, so the change in the reproduction number is negligible.

According to the sensitivity index of the state parameter *i*_*h*_ for the endemic equilibrium, the most influential model parameter for *i*_*h*_ is ψ_*h*_ (Table 7). The same parameter seems to be quite important for *l*_*h*_, as well. Another noteworthy parameter for *i*_*h*_ is μ_*x*_ and for both *i*_*h*_ and *l*_*h*_ is ν. At this point, it should be stressed that the three vine state variables of the fractional system, *s*_*h*_, *l*_*h*_ and *i*_*h*_, are expressed in relation to the maximum number of vines, *N*_*h*0_, which is a constant parameter. For this reason, when ψ_*h*_ changes, *s*_*h*_, *l*_*h*_, and *i*_*h*_ change in the same direction (increase or decrease simultaneously). Nevertheless, the reason why there is an increase in *s*_*h*_, *l*_*h*_, and *i*_*h*_ when ψ_*h*_ increases may be difficult to explain. The increase in the per capita replacement rate of vines would lead to an increase in the size of the vine population because the empty spots of the removed vines in the vineyard would be filled in. As a result, there would ultimately be an increase in all three vine stages related to a *Xff* infection. In situations such as the one analyzed here, i.e., when the disease is established in the population, most of this increase would end up mainly boosting the number of latent and infected vines. This would eventually mean that there are fewer healthy vines in relation to vines harboring *Xff* and, by extension, more infected GWSS, just as this model predicts (see *i*_*v*_ in relation to ψ_*h*_).

### Effectiveness of control strategies for regions with high PD pressure

Subgroups of current control strategies that share a mode of function are defined above. Table 8 links the parameters of this model with all the types of control strategies described in the first part of the review (Tables 1, 2).

**Table 8:** Overview of control strategies and the model parameters that represent them.

**Control strategies**	**Relevant parameters**
Control of insect vectors	*ψ_*v*_, μ_*v*1_, σ, β_*vh*_, β_*hv*_*
Control of non-vine host plants and vine propagation material	None due to model assumptions
Alteration to cropping techniques	*σ, μ_*x*_, γ*
Breeding or engineering PD-resistant/-tolerant *V. vinifera*	*β_*hv*_, γ*
Control via avirulent XYLEFA strains	*β_*hv*_, γ*
Control via other beneficial bacteria and fungi	*β_*hv*_, γ*
Bacteriophages of *Xff*	*β_*hv*_, μ_*x*_, γ*, and ***v***
Antagonistic bacteria	*β_*hv*_, μ_*x*_, γ*, and ***v***
Natural, antibacterial substances	*β_*hv*_, μ_*x*_, γ*, and ***v***
Other defense-stimulating compounds	*β_*hv*_, μ_*x*_, γ*, and ***v***

*Parameters σ, β_vh_, β_hv_, μ_x_, and v, shown by the sensitivity analysis to be key parameters for PD control, are in bold*.

Based on the results of the sensitivity analysis and on Table 8, it can be deduced that strategies reducing mainly σ, but also β_*vh*_ and β_*hv*_, could undermine an initial *Xff* spread. Therefore, GWSS control strategies linked with σ are crucial for reducing the incidence of PD in recently invaded areas. Such strategies are those impeding GWSS from feeding on vines or those rendering vines an unpleasant food source for GWSS. Existing strategies that target σ are screen barriers that protect vineyards from GWSSs, coating vines with kaolin and harpin-based products, and regulated deficit irrigation. A strategy that could decrease β_*vh*_ and β_*hv*_ is the release of major GWSS predators since their presence would reduce GWSS probing duration on vines. Parameter β_*vh*_ could also be minimized *via* paratransgenesis, while β_*hv*_ appears to be affected by many more strategies. By definition, these involve all prophylactic strategies that breed or engineer resistance/tolerance in vines and that control *Xff* using avirulent XYLEFA strains, beneficial bacteria and fungi (i.e., pre-infection stage). Furthermore, β_*hv*_ is affected by all therapeutic strategies (i.e., post-infection stage). As regards *N*_*h*0_ and μ_2*v*_, the analysis demonstrated their negative impact on *Xff* transmission. Unfortunately, *N*_*h*0_ and μ_2*v*_ are parameters over which there is no control as they are determined by land availability and vine productivity, and GWSS population dynamics, respectively.

These results, though, combined with the literature review indicate a positive connection between mainly ψ_*h*_, but also *v* and the prevalence of PD in a vine population. Moreover, a decrease in μ_*x*_ is partially responsible for the eventual successful establishment of *Xff*. Parameter ψ_*h*_ cannot be managed by a PD control strategy since it strictly depends on the budget of vine-growers and nurseries' responsiveness. Evidently, a strategy directly reducing μ_*x*_ is the roguing of vines that have developed persistent PD. Parameter *v* does not relate to any strategy of breeding or engineering resistance/tolerance in vines, or strategies that control *Xff* using avirulent XYLEFA strains, beneficial bacteria and fungi. This is correct, given that any preventive strategy is not applicable when the vine reaches the symptomatic stage. Conversely, both *v* and μ_*x*_ can be controlled by all the therapeutic strategies mentioned.

Overall, this model shows that all studied therapeutic strategies affect three key parameters: β_*hv*_, μ_*x*_, and *v*. These parameters appear to contribute by blocking an initial spread of PD and are also extremely promising against *Xff* establishment in a vine population. Hence, it is suggested that research on and application of those strategies need to be prioritized. For areas in which *Xff* is not yet persistent in the majority of vines, vine-growers are advised to principally employ strategies that reduce GWSS preference for vines and vine accessibility. Lastly, the model suggests regular inspection of vineyards to monitor PD symptom severity and expeditious removal of problematic vines. This mode of management may significantly impede *Xff* transmission in a healthy vine population.

## General conclusions

There has been a systematic effort to reduce the presence of *Xff* in vineyards for more than 20 years. To that end, a plethora of approaches has been studied. Considering that the threat of XYLEFA-associated diseases is now increasingly widespread, researchers need to direct their attention toward improving those prophylactic strategies that have already been successful in vineyards. Furthermore, future studies should also focus on therapeutic strategies, such as successful field application of bacteriophage cocktails or other antagonistic microorganisms, since curing infected vines appears to be vital for the survival of the wine industry in PD-demarcated areas. This statement is supported by the results of the numerical simulation of the developed model. It is proposed that alternative strategies, should be designed only when already promising control strategies consistently fail to render the expected results in vineyards. In such an event, researchers should consider the importance of the parameters determined by this model as being crucial to the initial spread and establishment of *Xff* in a healthy vine population. Nonetheless, it should also be borne in mind that the evaluation of key parameters here was based on a model that considered SC, which is a region facing high PD pressure. Shifts regarding the importance of different parameters are to be expected in areas where PD epidemiology has different characteristics.

## Author contributions

IK conceived the idea, performed the literature search and collection of data, wrote the review part, designed figures and the model, performed the calculations, contributed to writing the modeling part, and interpreted the results. TP designed the model and the computational framework, ran the model, performed the calculations and analyses, designed figures, contributed to writing the modeling part. LE-J, M-FS, and LH discussed the results and reviewed the manuscript. M-FS and LH were in charge of overall direction and planning.

### Conflict of interest statement

The authors declare that the research was conducted in the absence of any commercial or financial relationships that could be construed as a potential conflict of interest.

## Footnotes

^1^Readers of this review should be warned that Rolshausen et al. have significantly relied on the Proceedings of Pierce's Disease Research Symposia to publish updates of their project on fungal anti-*Xff* endophytes. The purpose of this review is to offer the readers a complete and detailed overview of all attempts to control *Xff*. Thus, even if not always peer-reviewed, the authors believe that overlooking this project from the review would be reductive.

## Abbreviations

XYLEFA: *Xylella fastidiosa*
*Xff*: *Xylella fastidiosa* subsp. *fastidiosa*
PD: Pierce's disease
GWSS: glassy-winged sharpshooter
SC: Southern California.
